# Efficient production of mannosylerythritol lipids by a marine yeast *Moesziomyces aphidis* XM01 and their application as self-assembly nanomicelles

**DOI:** 10.1007/s42995-022-00135-0

**Published:** 2022-06-27

**Authors:** Guanshuo Yu, Xiaoxiang Wang, Chao Zhang, Zhe Chi, Zhenming Chi, Guanglei Liu

**Affiliations:** 1 College of Marine Life Sciences, Ocean University of China, Qingdao, 266003 China; 2 Laboratory for Marine Biology and Biotechnology, Pilot National Laboratory for Marine Science and Technology (Qingdao), Qingdao, 266237 China

**Keywords:** Mannosylerythritol lipids, Two-stage fed-batch fermentation, Self-assembly nanomicelles, Antimicrobial activity, Drug encapsulation and release

## Abstract

Mannosylerythritol lipids (MELs) are one of the most promising biosurfactants because of their excellent physicochemical properties, high environmental compatibility, and various biological functions. In this study, a mangrove yeast strain *Moesziomyces aphidis* XM01 was identified and used for efficient extracellular MEL production. The MEL titer reached 64.5 ± 0.7 g/L at flask level within 7 days with the optimized nitrogen and carbon source of 2.0 g/L NaNO_3_ and 70 g/L soybean oil. Furthermore, during a 10-L two-stage fed-batch fermentation, the final MEL titer reached 113.6 ± 3.1 g/L within 8 days, with prominent productivity and yield of 14.2 g·L^−1^·day^−1^ and 94.6 g/g_(glucose and soybean oil)_. Structural analysis indicated that the produced MELs were mainly MEL-A and its fatty acid profile was composed of only medium-chain fatty acids (C8–C12), especially C10 acids (77.81%). Further applications of this compound were evaluated as one-step self-assembly nanomicelles. The obtained MEL nanomicelles showed good physicochemical stability and antibacterial activity. In addition, using clarithromycin as a model hydrophobic drug, the MEL nanomicelles exhibited high loading capacity and could be used for the controlled and sustained drug release in low-pH environments. Therefore, *M. aphidis* XM01 is an excellent candidate for efficient MEL production, and the prepared MEL nanomicelles have broad application prospects in the pharmaceutical and cosmetic fields.

**Supplementary Information:**

The online version contains supplementary material available at 10.1007/s42995-022-00135-0.

## Introduction

Mannosylerythritol lipids (MELs) are designated as a class of glycolipids consisting of the partially acylated derivatives of 4-O-β-d-mannopyranosyl-d-erythritol as the hydrophilic group and a variety of fatty acids as the hydrophobic chains (Coelho et al. 2020). On the basis of the different location and number of acetylation, MELs are classified as MEL-A (diacetylated at mannosyl C‐4 and C‐6), MEL-B (mono-acetylated at mannosyl C-6), MEL-C (mono-acetylated at mannosyl C-4), and MEL-D (deacetylated) (Saika et al. 2018). So far, MEL production is predominantly reported in basidiomycetous yeasts of the genera *Ustilago* and *Pseudozyma* (Konishi et al. 2007, 2008; Morita et al. 2007; Saika et al. 2018). These produced MELs are reported to exhibit different structural compositions and properties, which are attributed to various yeast strains, as well as diverse carbon source and culture conditions.

Glycolipids can be used as biosurfactants with unique properties compared to chemical counterparts, such as high environmental compatibility, mild production conditions, and various biological functions (Coelho et al. 2020; Valotteau et al. 2020). In this regard, MELs are one of the most promising glycolipids. MELs possess excellent physicochemical properties, including good emulsification and low critical micelle concentration (CMC) (Morita et al. 2009), and also versatile biological properties, including antibacterial and antibiofilm activity against foodborne gram-positive *Staphylococcus aureus* (Shu et al. 2020), moisturizing, antioxidant, and protective effects in skin cells (Coelho et al. 2020), differentiation-inducing activities against human leukemia cells, mouse melanoma and pheochromocytoma cells (Yu et al. 2015), and inhibiting the secretion of inflammatory mediators from mast cells (Morita et al. 2011). Moreover, immunosuppressive, immunomodulating, antitumor, and anti-inflammatory activities have also been emphasized for MELs as previously described (Coelho et al. 2020). Therefore, the production and application of MELs show significant biotechnological potential and deserve to be further developed and studied.

In general, amphiphilic molecules are able to naturally self-assemble into hierarchically ordered nanoassemblies by the intermolecular interaction of hydrophobic force, hydrogen bonds, and van der Waals force (Sekhar et al. 2018). Thus, in addition to excellent surface activity, MELs also can be developed as functional materials and/or systems through their self-assembling properties (Kitamoto et al. 2009). A previous study demonstrated that the MELs produced from *Ustilago maydis* CGMCC 5.203 could be used for the synthesis of gold nanoparticles which showed potential anticancer, antioxidant activity, and antibacterial activity (Bakur et al. 2019). In addition, MEL-A is also related to the improvement of DNA and siRNA transfection efficiency in cationic liposome systems (Inoh et al. 2011). However, there is still limited available information on physicochemical properties and practical applications of MEL self-assembly nanomicelles.

In the present study, a mangrove yeast *Moesziomyces aphidis* XM01 was isolated, identified, and employed for efficient MEL production by a two-stage fed-batch bioprocess. The produced MELs were characterized and further used for the preparation of self-assembly nanomicelles. Finally, the physicochemical stability, antimicrobial activity, drug encapsulation, and release properties of MEL nanomicelles were evaluated, indicating their potential application in medical, pharmaceutical, and cosmetic fields.

## Results and discussion

### Identification of the marine yeast XM01 reveals its potential as a MEL-A producer

**Fig. 1:**
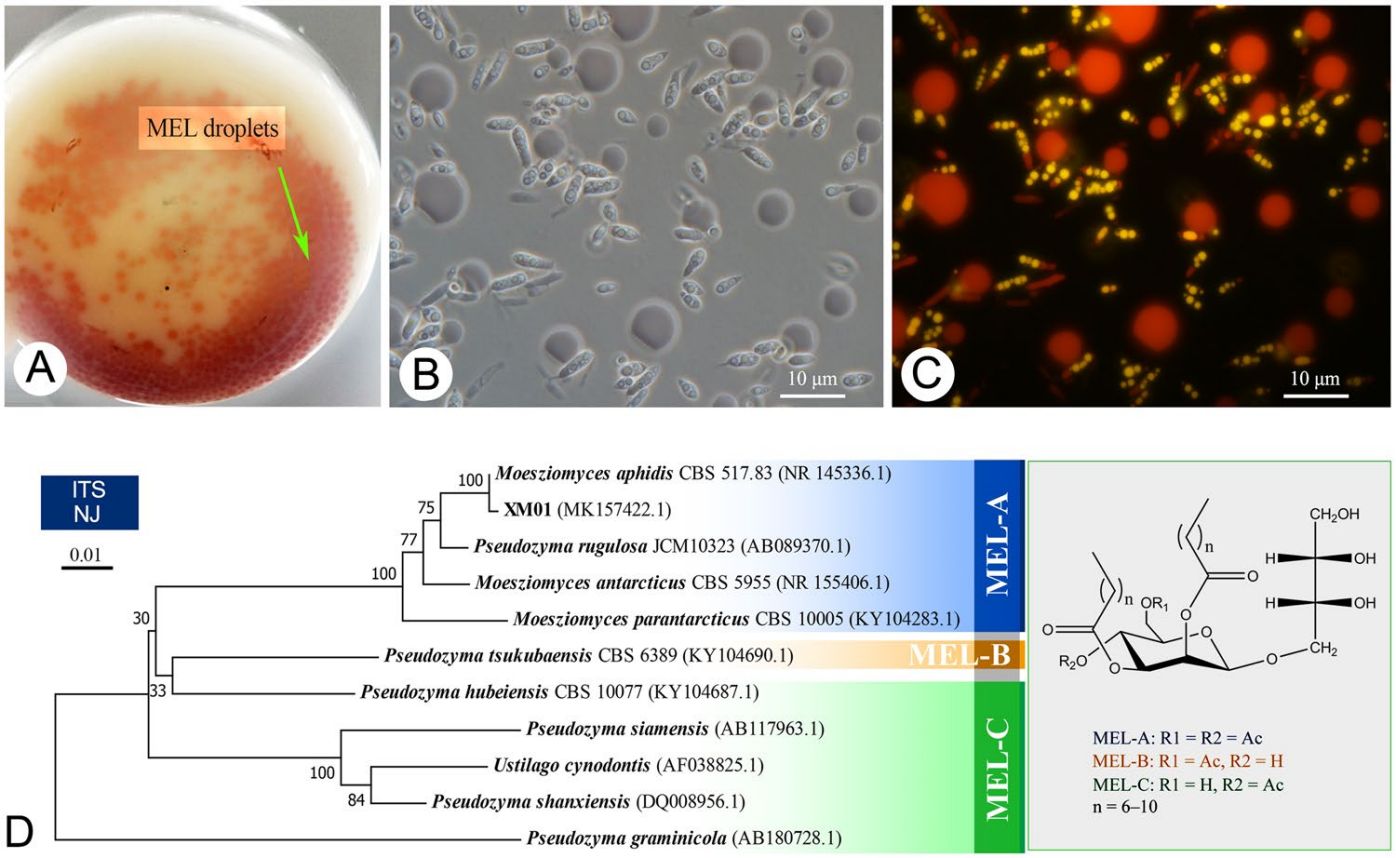
Identification of a MEL producing yeast XM01. **A** The MEL droplets (red) secreted by the strain XM01 in the culture. Intracellular lipid particles (yellow ones) and extracellular MEL particles (red ones) produced by the strain XM01 were observed under a phase-contrast microscope (**B**) and a fluorescence microscope (**C**). **D** The phylogenetic tree associated with MEL production pattern of the strain XM01 and other yeast relatives based on neighbor-joining analysis of ITS sequences. Bootstrap values at the notes are from 1000 replicates

A newly isolated marine strain XM01 from mangrove systems in Hainan Province of China was used to produce glycolipids from soybean oil. As a result, the strain XM01 was found to secrete numerous red and hydrophobic droplets (Fig. 1A) with a titer of 59.8 g/L at 168 h, indicating its potential for glycolipids production. Subsequently, the molecular identification indicated that the ITS sequence of the strain XM01 (NCBI Accession number: MK157422.1) exhibited 99.06% similarity to that of the type strain *Moesziomyces aphidis* CBS517.83. Thus, as the topology of the phylograms in Fig. 1D confirmed, the yeast strain XM01 belonged to the species *M. aphidis*. Generally, the taxonomic character of the producers and the production pattern of glycolipids behaved in excellent correlation (Konishi et al. 2007). Specifically, *Pseudozyma antarctica*, *P. parantarctica*, and *P. rugulosa* are known to produce large quantities of MEL-A together with MEL-B, MEL-C, and MEL-D as minor components (Morita et al. 2007). *P. tsukubaensis* and *P. crassa* have been reported to produce predominantly MEL-B (Saika et al. 2018). For MEL-C, *P. shanxiensis*, *P. hubeiensis*, *P. siamensis*, and *P. graminicola* are main producers (Konishi et al. 2008). Therefore, the topology of the phylogenetic tree associated with MEL production pattern in Fig. 1D suggests that the strain XM01 may be a MEL producer with a main component of MEL-A.

In addition, the morphologic investigation of the XM01 culture under fluorescence microscopy via Nile red staining revealed that the yeast cells were cylindrical to fusiform with intracellular yellow fluorescent oil droplets and extracellular red fluorescent MEL molecules (Fig. 1B, C). In contrast, MEL produced by *P. rugulosa* NBRC 10,877 appeared as a strong orange-red fluorescence using Nile Red as a lipid probe, indicating this strain intracellularly accumulated MEL during cultivation (Morita et al. 2006). *P. antarctica* T-34 also significantly accumulates MEL in the cells (Kitamoto et al. 1992). These results imply that the strain XM01 could efficiently transport the synthetic MELs outside the cell. Therefore, the strain XM01 was not only a valuable candidate for MEL production but can also help to minimize cytotoxicity and simplify the extraction process due to the non-intracellular accumulation nature of MEL.

### Physiological alkaline nitrate and soybean oil are suitable for MEL production by XM01

**Fig. 2:**
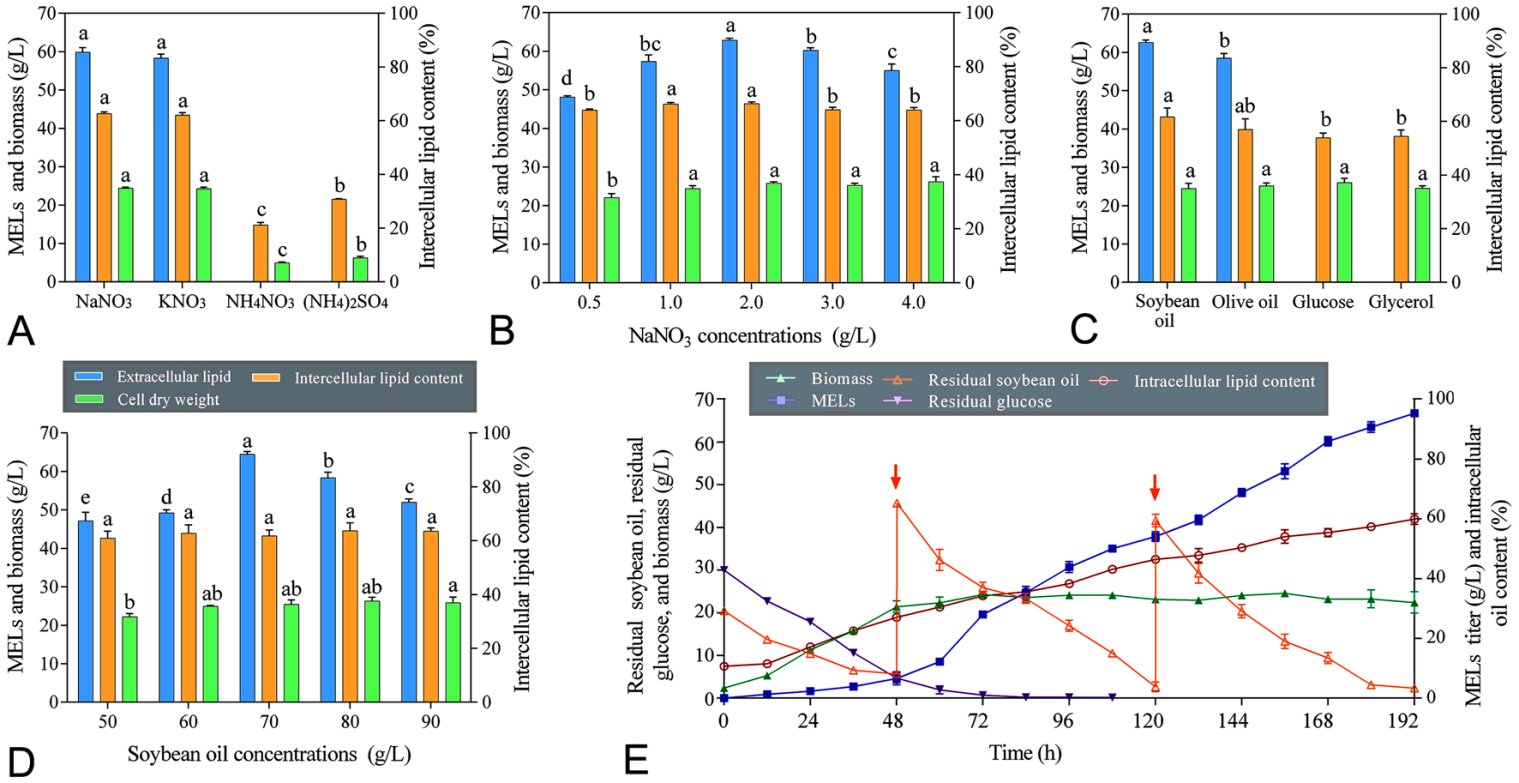
Optimization of the nitrogen and carbon sources for MEL biosynthesis and MEL production by 10-L fermentation. Effects of different types of nitrogen sources (**A**), different concentrations of NaNO_3_ (**B**), different types of carbon sources (**C**), different concentrations of soybean oil (**D**) on the MEL production, intracellular lipid accumulation, and cell growth. The values are the means of three independent determinations. Values with different superscript letters differ significantly and are determined statistically by one-way ANOVA, Tukey, *P* < 0.05. (**E**) The time course of the MEL production, intracellular lipid accumulation, biomass, residual glucose and residual soybean oil during 10-L fermentation by the strain XM01. The values are the means of three independent determinations

Previous investigations showed that nitrogen and carbon sources are essential parameters for MEL production (Konishi et al. 2008; Niu et al. 2019). Thus, to further improve the fermentation conditions of the strain XM01, the effects of different types and concentrations of nitrogen and carbon sources on MEL production, intracellular lipid accumulation, and cell growth were investigated. As indicated in Fig. 2A, both the physiological alkaline salts of NaNO_3_ and KNO_3_ gave high MEL titers of nearly 60 g/L. However, considering the cost factor, NaNO_3_ was selected as the optimum nitrogen source, and the highest MEL yield was obtained from NaNO_3_ with an optimum concentration of 2.0 g/L (Fig. 2B). In addition, it is noteworthy that both the physiological acid salts of NH_4_NO_3_ and (NH_4_)_2_SO_4_ gave no MEL production, and intracellular lipids accumulation and cell growth were also significantly impaired. A similar effect was also observed in the studies of *P. hubeiensis* KM-59 (Konishi et al. 2008) and *P. rugulosa* NBRC 10877 (Morita et al. 2006). To investigate this, the pH and lipase activity during the fermentation were measured. As indicated in Supplementary Fig. S1, both the pH and lipase activity of the (NH_4_)_2_SO_4_ group was significantly lower than those of the NaNO_3_ group. This result suggests that in the media containing physiological acid salts, the consumption of ammonium ion can lead to the decrease of pH and then cause the impairment of lipase activity, thereby impeding soybean oil assimilation and MEL production.

Effects of carbon sources on MEL production were also examined at 28 °C for 7 days. As indicated in Fig. 2C and D, both soybean oil and olive oil gave high MEL titers, and the highest yield was obtained from soybean oil with an optimum of 70.0 g/L. In contrast, glucose and glycerol could only be used for cell growth and intracellular lipid production, but not for MEL production. These results suggest that the de novo fatty acids synthesis pathway followed by β-oxidation has little contribution to MEL synthesis in *M. aphidis* XM01, and the medium-length fatty acids of MEL are derived from the β-oxidation intermediates of the substrates supplied. This is probably the reason that β-oxidation is subject to glucose repression (Ramirez and Lorenz 2009). However, the detailed regulatory mechanism is still unknown. Notably, as shown in Fig. 2D, the MEL titer reached 64.5 ± 0.7 g/L within 7 days, which was more than those of the other MEL producers at flask level reported so far, such as *P. antarctica* (34 g/L from 8% soybean oil (v/v) within 7 days) (Kitamoto et al. 1992), *P. aphidis* DSMZ 70725 (36 g/L from 67 g/L soybean oil within 7 days) (Rau et al. 2005a), and *P. antarctica* (40 g/L from 67 g/L soybean oil within 7 days) (Morita et al. 2007), suggesting the potential application of XM01 in the industrial production of MELs.

### Efficient MEL production by *M. aphidis* XM01 using a two-stage fed-batch bioprocess

According to previous literature (Konishi et al. 2008; Rau et al. 2005a), fed-batch cultivation is one of the most effective methods for MEL production. In this study, a two-stage bioprocess, namely the first stage of cell growth and the second stage of MEL fed-batch fermentation, was employed in a 10-L fermenter. To improve biomass at the first stage, the concentration of soybean oil was reduced to 20 g/L and 20 g/L of glucose was supplied in the initial fed-batch medium. As indicated in Fig. 2E, the biomass and utilization of total sugar increased to 21.3 ± 1.4 g/L and 93.0 ± 1.5% in 48 h, respectively. This result suggests that cells have entered the stationary phase, and that glucose is almost assimilated thereby avoiding the appearance of glucose repression. In the fed-batch second stage, 30 g/L additional soybean oil was added into the fermentor at 48 and 120 h of the cultivation. As expected, the MEL titer was raised dramatically to achieve a maximum of 91.7 ± 2.1 g/L within 192 h, with the final biomass, residual soybean oil, and intracellular lipid content of 22.4 ± 2.5 g/L, 2.3 ± 0.9 g/L and 60.0 ± 1.8%, respectively (Fig. 2E). Therefore, the MEL titer was recalculated and the result was now 113.6 ± 3.1 g/L.

Previously, several successful attempts were made to produce MELs using *Pseudozyma* strains at the bioreactor level, indicating the MELs titers ranging from 76 to 165 g/L (Kitamoto et al. 2001; Konishi et al. 2008; Morita et al. 2007, 2015; Rau et al. 2005a, b). However, it is noteworthy that all these reported processes involve a long fermentation period of 12–28 days, resulting in a high risk of microbial contamination, high production costs of energy and substrate, and relatively low productivities ranging from 5.0 to 13.9 g·L^−1^·day^−1^ (Kitamoto et al. 2001; Konishi et al. 2008; Morita et al. 2007, 2015; Rau et al. 2005a, b). By contrast, the fermentation period in this study was 8 days with productivity and yield of 14.2 g·L^−1^·day^−1^ and 94.6 g/g_(glucose and soybean oil)_, suggesting that the production by *M. aphidis* XM01 using a two-stage fed-batch bioprocess was a prominent candidate for industrial production of MELs.

### Characterization of the MELs produced by *M. aphidis* XM01

Each yeast produced a mixture of structurally similar MELs, differing in the degree of acetylation in mannose, the chain length, and saturation variants of fatty acids (Coelho et al. 2020). In this study, TLC analysis of the crude and purified content from the batch fermentation revealed that the MELs produced by the strain XM01 were a mix of MELs -A, -B and -C, and MEL-A was the main component (Supplementary Fig. S2A). Furthermore, the type of polyols and degree of acetylation of the MELs were determined by HPLC. According to the retention time of the standard substances, mannose, erythritol, and acetic acid were detected with a molar ratio of 1:1.11:1.78 (Supplementary Fig. S2B). Considering that the acetylation degree of MELs -A, -B, -C and -D were 2, 1, 1 and 0, respectively, the average acetylation degree of 1.78 in the mannose further confirmed that the strain XM01 produced predominantly MEL-A, which was consistent with our postulation on the molecular phylogenetic tree.

To further investigate the structural composition of the major component MEL, MEL-A was purified using a silica gel column as described in “Materials and methods” and applied to GC/MS and LC–ESI–MS analysis. As indicated in Table 1, the fatty acids in MEL-A produced from soybean oil were C8:0, C8:1, C10:0, C10:1, C12:0, and C12:1 acids, and the dominant acids were C10:0 (38.02%) and C10:1 (39.07%). This result was consistent with the observation from LC–ESI–MS (Supplementary Fig. S2C) that all the six types of fatty acid chains listed in the GC–MS result (Table 1) were identified, and that the Peak 4 (C_34_O_13_H_58_, *m*/*z*) with the fatty acid chains of C10:0 and C10:1 showed the highest relative abundance. The intracellular lipids produced by strain XM01 were also analyzed by GC/MS. As indicated in Table 1, in addition to the dominant fatty acids of C16:0, C18:0, C18:1, C18:2 and C18:3 similar to other oleaginous yeast lipids reported (Shi et al. 2018; Wang et al. 2019), the fatty acid precursors of MEL including C8, C10, and C12 were also detectable.

**Table 1:** Fatty acid composition of soybean oil, and the intracellular lipids and MEL-A produced by *M. aphidis* XM01

Fatty acid chain	Composition (%)		
	Soybean oil	MEL-A	Intracellular lipids
Octanoic acid (C8:0)	–	16.22	0.42
4-Octenoic acid (C8:1)	–	4.51	–
Decanoic acid (C10:0)	–	38.02	1.13
4-Decenoic acid (C10:1)	–	39.79	0.75
Dodecanoic acid (C12:0)	–	0.73	1.05
5-Dodecenoic acid (C12:1)	–	0.73	–
Tetradecanoic acid (C14:0)	0.18	–	0.24
Hexadecanoic acid (C16:0)	15.54	–	7.68
9-Hexadecenoic acid (C16:1)	0.16	–	0.14
Octadecanoic acid (C18:0)	7.55	–	3.10
9-Octadecenoic acid (C18:1)	29.44	–	38.19
9,12-Octadecadienoic acid (C18:2)	36.07	–	44.09
9,12,15-Octadecatrienoic acid (C18:3)	8.29	–	3.22
Eicosanoic acid (C20:0)	0.80	–	0.02
11-Eicosenoic acid (C20:1)	0.48	–	–
Docosanoic acid (C22:0)	1.12	–	–
Tetracosanoic acid (C24:0)	0.37	–	–

Similar to the strain XM01, the previously reported high-level MEL-A producers such as *P. aphidis*, *P. antarctica*, *P. rugulosa*, and *P. parantarctica* produce mainly MEL-A together with MEL-B and MEL-C as minor components (Konishi et al. 2007; Niu et al. 2019; Rau et al. 2005a). More significantly, the major fatty acid chains of the obtained MEL-A from soybean oil are C8 and C10 acids. However, their minor components of fatty acid profiles are different due to the genetic diversification of different strains. For example, in addition to C8 and C10 acids, MELs from *P. aphidis* ZJUDM34 grown on soybean oil consists of C6:0 (23.19%), C18:1 (7.37%), and C18:2 (9.57%) (Niu et al. 2019). In another study, C14 acids were detectable with the compositions ranging from 0.8 to 7.7% in all the tested MEL-A samples from ten *Pseudozyma* strains (Konishi et al. 2007). In contrast, the fatty acid profile of the MEL-A in this study was composed of only medium-chain fatty acids (C8–C12), especially C10 acids (77.81%). This suggested that though the fatty acids derived from the carbon source supplied various fatty acid chains ranging from C14–C24 (Table 1), the mainly β-oxidation intermediates used for MEL synthesis were C8–C12 and mainly C10 in the strain XM01. Therefore, the strain XM01 was a valuable microbial cell factory for the production of medium-chain fatty acids, which has been regarded as functional lipids for the prevention and treatment of metabolic syndrome, such as type 2 diabetes (Nagao and Yanagita 2010).

### MELs can be used for the preparation of one-step self-assembly nanomicelles

As stated above, the MELs produced by the strain XM01 were composed of mannose and erythritol as a hydrophilic moiety and medium-chain fatty acids as a hydrophobic moiety. This amphiphilic property prompts the MELs to form highly organized structures via self-assembly, thereby developing their new functional structures and systems. To address this, the self-aggregation behavior of the MELs produced by the strain XM01 was analyzed by spectrofluorimetry using a pyrene hydrophobic probe. As indicated in Supplementary Fig. S3A, a plot of the ratios of *I*_373_/*I*_384_ (*I*_1_/*I*_3_) against the logarithmic concentration showed a single step change indicating the presence of a single type of aggregate. The critical aggregation concentration (CAC), which was defined as the threshold concentration of self-aggregation of polymeric amphiphiles, was determined to be 0.79 mg/L, and approximately 1.12 × 10^–3^ mmol/L which was calculated based on the molecular weight of the main component MEL-A (C10:0 and C10:1) (Supplementary Fig. S2C).

Subsequently, MEL micelles were fabricated by one-step self-assembly using ultrasonic treatment and were confirmed by the detection of the Tyndall effect (Supplementary Fig. S3C). Furthermore, the dynamic light scattering (DLS) experiments showed the average diameter, polydispersity index, and zeta-potential of MEL micelles of 94.26 ± 1.63 nm, 0.14 ± 0.03, and − 27.53 ± 1.73, respectively. Furthermore, the morphological features of MEL aggregates by transmission electron microscopy (TEM) analysis confirmed the spherical shape of nanomicelles (Supplementary Fig. S3B). They were found to be non-aggregated and uniformly distributed over the TEM grid which was consistent with the size distribution from DLS measurements (Supplementary Fig. S3D) and further supports the homogeneity of the MEL nanomicelles.

### MEL nanomicelles show good physicochemical stability

Physicochemical stability is an important factor that limits the practical applications of MEL nanomicelles in various fields. Therefore, the stability of MEL nanomicelles was investigated in this study. For time-dependent aggregation behavior, as clearly observed from Supplementary Fig. S4A, MEL nanomicelles exhibited an excellent stability at 4 °C because they could maintain the constant particle size of approximately 90 nm and polydispersity index (PDI) ranging from 0.15–0.2 for 35 days. Importantly, when the temperature was switched to 25 °C, these MEL nanomicelles could keep their morphologies for 21 days, and during the subsequent week, the particle size only increased slightly while the polydispersity index still remained stable. This suggests that MEL nanomicelles could achieve excellent storage stability at room temperature for one month, similar to that at refrigeration, which is beneficial to saving energy and protecting the environment.

Furthermore, to understand the thermal behavior of MEL nanomicelles in solution, a DSC experiment was carried out. Compared with the MEL solution, MEL nanomicelles did not show any thermal response from 20 to 80 °C (Supplementary Fig. S4B), confirming that these micelles are thermally stable. For pH stability, as shown in Fig. S4C, the particle size of MEL nanomicelles was in the range of 100–200 nm at pH 4.0–11.0, and the PDI values of these groups were lower than 0.3, indicating the colloidal stability of MEL nanomicelles at pH 4.0–8.0. However, when the pH was less than 3.0, the particle size increased significantly. In fact, a similar result was noticed in visual observation. As indicated in Supplementary Fig. S4D, the solution was transparent when the pH was more than 3.0, and an obviously turbid appearance was observed at pH 1.0–3.0. Notably, the zeta-potentials of MEL nanomicelles were close to zero at pH 2.0–3.0 and were even positively charged at pH 1.0, suggesting that the net surface charge of MEL nanomicelles reduced at low pH, resulting in the weakened repulsion between particles. Similarly, another reported zein/sophorolipid nanoparticles show colloidal instability at pH 2.0–4.0 (Yuan et al. 2021).

Generally, nanomicelles are regarded as the best pharmaceutical carriers for hydrophobic drugs (Bose et al. 2021). In this study, the self-assembly MEL nanomicelles showed good physicochemical stability, which allows the nanomicelles to retain the integrity and maintain its drug content till the time it reaches its target site. Moreover, the low-pH sensitivity of MEL nanomicelles is of benefit to the controlled release of loaded drugs. Thus, MEL nanomicelles will attract much attention in medical and pharmaceutical fields.

### MEL nanomicelles show broad-spectrum antibacterial activity

Microbial biosurfactants are biobased compounds and have attracted the interest of the medical industry for the development of new medicines due to their antimicrobial properties (Valotteau et al. 2020). MELs also exhibit antimicrobial properties due to their damaging effect on the bilayer cell membranes of microorganisms (Fukuoka et al. 2007). However, there is little research on the antimicrobial activity of MEL nanomicelles. In this study, the minimum inhibitory concentrations (MIC) of the prepared MEL nanomicelles were determined as 0.5, 1.0, 0.5, and 2.0 mg/ml towards four pathogenic bacteria of *Staphylococcus aureus*, *Staphylococcus epidermidis*, *Pseudomonas aeruginosa*, and *Escherichia coli*, respectively (Supplementary Fig. S5A), confirming the antibacterial ability of MEL nanomicelles. Furthermore, it is noteworthy that the reported MIC of MELs toward *S. aureus* is 0.625 mg/ml (Shu et al. 2020), which is slightly higher than that of the MEL nanomicelles prepared in this study (0.5 mg/ml). In a previous study (Nashida et al. 2018), a series of homologs of MELs obtained by chemical synthesis were used for the evaluation of the influence of alkyl chain length on the antimicrobial performance of MELs. The results indicated 8 and 10 carbons exhibited significantly higher antimicrobial activity than the compounds with 6, 12, and 14 carbons. Therefore, the high proportion of C8 and C10 fatty acid chain in the MELs of this study (Table 1, the total proportion of C8 and C10 was 98.54%) conferred their good antimicrobial performance. In addition, it has been reported that MELs show a better antibacterial performance against Gram-positive bacteria (Coelho et al. 2020), such as *S. aureus* (Shu et al. 2020) and *Bacillus cereus* (Ceresa et al. 2020; Shu et al. 2019). However, the results in Supplementary Fig. S5A indicated that the prepared MEL nanomicelles also showed a low MIC towards a Gram-negative bacteria of *P. aeruginosa* (0.5 mg/ml). Therefore, MEL nanomicelles show a broad antibacterial spectrum, which makes it possible to be used as antimicrobial agents. In addition, because of their own antibacterial activity, MEL nanomicelles can be used for the preparation of multifunctional nanomicelles after drug loading.

### Encapsulation and release of a hydrophobic drug

Hydrophobe-loaded nanoparticles have shown immense potential in the field of nanomedicines and agrochemicals (Sekhar et al. 2018). In this section, clarithromycin (CLR), a macrolide antibiotic used for a wide variety of mild-to-moderate bacterial infections, was employed as a model hydrophobic drug to understand the drug loading efficiency and drug release behavior of MEL nanomicelles. The amount of MEL nanomicelles was fixed, and the initial clarithromycin input varied from 10 to 40% (w/w) of the MEL weight. As indicated in Supplementary Fig. S5B, when the initial clarithromycin input was changed from 10 to 30%, there were no significant difference in their encapsulation efficiency (EE) values. However, when the initial clarithromycin input was raised to 40%, the EE value decreased significantly, suggesting that 30% was the optimum initial drug input to load clarithromycin for the maximum capacity of the MEL nanomicelles. Meanwhile, the loading capacity (LC) values increased significantly (*P* < 0.05) until the initial clarithromycin input was 30%. Based on the above results, the optimized clarithromycin input was 30% (w/w) with a EE value of 79.7% and an LC value of 23.9%. In addition, it was noteworthy that under this optimum condition, the particle sizes of the clarithromycin-loaded nanomicelle (MEL/CLR) were 123.79 ± 2.15 nm which was significantly higher than that of the empty MEL nanomicelles (Supplementary Fig. S5C), indicating that encapsulated clarithromycin led to larger particle sizes. Furthermore, the PDI value (0.23) of MEL/CLR was still low and its zeta-potential (− 28.37 ± 1.81) was similar to the empty MEL nanomicelles, suggesting the prepared MEL/CLR was stable and could be used for the following studies.

In a previous study, carboxymethyl chitosan was hydrophobically modified with stearic acid, and then was conjugated with urea to acquire U-CMCS-g-SA co-polymers (Cong et al. 2019). The clarithromycin loading capacity of this nanomicelle (24.5%) is slightly higher than that of the MEL nanomicelles in this study (23.9%). However, the two loading capacity values are nearly twice as much as that of a poly(lactic-co-glycolic acid) cored nanoparticle (12.4%) with a initial clarithromycin input of 30% (Angsantikul et al. 2018), which was likely ascribed to the stronger hydrophobic interaction between clarithromycin and fatty acid chains. These results suggest that the MEL nanomicelles fabricated by one-step self-assembly in this work had good encapsulation properties.

In consideration of the low-pH sensitivity (Supplementary Fig. S4C), the MEL nanomicelles could be used for the hydrophobic drug release in acid environments, such as gastric fluid. Therefore, the release kinetic of clarithromycin from MEL/CLR in simulated gastric fluid (SGF pH 1.2) was examined. As indicated in Supplementary Fig. S5D, the loaded clarithromycin could be sustainedly released in the SGF within 24 h. Specifically, an approximately 91.0% cumulative release of clarithromycin was determined in 24 h, while approximately 37.1% of clarithromycin was released from the nanomicelles in 2 h. However, for the control group in PBS buffer (pH 7.4), only 9.7% of clarithromycin was released, suggesting that the good stability of MEL nanomicelles in neutral environments (Supplementary Fig. S4) could efficiently prevent the leakage of encapsulated drugs. These results confirm that the MEL nanomicelles could be used for the controlled and sustained drug release in low-pH environments, suggesting their application potential in medical and pharmaceutical fields. In addition, the alkyl chain length and hydrophilic headgroup of glycolipids are illuminated to be important factors that impact the hydrophobic drug encapsulation and release of glycolipid nanomicelles (Sekhar et al. 2018). Therefore, the customized MEL production from the engineered yeast cells with rationally designed MEL biosynthetic pathway will be a future research direction to prepare unique nanomicelles.

## Conclusion

In this study, a mangrove yeast strain *M. aphidis* XM01 showed the promising ability of MELs efficient production. During a 10-L two-stage fed-batch fermentation, the final MEL titer reached 113.6 ± 3.1 g/L within 8 days, with prominent productivity and yield of 14.2 g·L^−1^·day^−1^ and 94.6 g/g_(glucose and soybean oil)_, indicating that the *M. aphidis* XM01 strain could be used as an alternative microbial cell factory for industrial production of MELs from soybean oil. Structural analysis revealed that the MELs produced by the strain XM01 were composed of mannose and erythritol as a hydrophilic moiety and mainly C8 and C10 medium-chain fatty acids and could be used for the preparation of one-step self-assembly nanomicelles. The obtained MEL nanomicelles showed good physicochemical stability, antibacterial activity, and encapsulation and release behavior. These properties together with the biodegradability, the low toxicity, and the feasibility of high-level microbial production, make MEL nanomicelles attractive across the medical, pharmaceutical, and cosmetic fields.

## Materials and methods

### Yeast strain and cultivation

The marine yeast strain XM01 used in this study was isolated from mangrove plants in Hainan, China. For MEL production, the seed medium consisted of 30.0 g/L glucose, 1.0 g/L NH_4_NO_3_, 0.3 g/L KH_2_PO_4_, and 1.0 g/L yeast extract, and the MEL production medium consisted of 80 g/L soybean oil, 3.0 g/L NaNO_3_, 0.2 g/L KH_2_PO_4_, 0.2 g/L MgSO_4_·7H_2_O, and 1.0 g/L yeast extract (Morita et al. 2006). The optimized MEL production medium was composed of 70 g/L soybean oil, 2.0 g/L NaNO_3_, 0.2 g/L KH_2_PO_4_, 0.2 g/L MgSO_4_·7H_2_O, and 1.0 g/L yeast extract.

### Molecular identification of the strain XM01

The internal transcribed spacer region (ITS) of the rRNA gene cluster was amplified using the common primers ITS1 and ITS4 and sequenced as described by Wang et al. (2019). The ITS sequence obtained above was aligned using BLAST analysis (http://blast.ncbi.nlm.nih.gov/Blast.cgi). Multiple alignment and phylogenetic tree construction of *ITS* sequences were performed by the Mega 7 software (Kumar et al. 2016).

### Microscopic visualization of MEL and intracellular lipid

MEL and intracellular lipid were visualized by the method described previously (Wang et al. 2019). Briefly, after the XM01 strain was cultured in the MEL production medium for 168 h, the culture was mixed with 5 μg/ml Nile red in DMSO and incubated for 10 min. Visualization was performed under the fluorescence mode with the excited and the emitted wavelength of 530 and 626 nm at 100× magnification using an Olympus U-LH100HG fluorescent microscope.

### Effect of nutritional parameters on MEL production

The yeast strain XM01 was cultivated in the seed medium at 28 °C for 1 day. Then, 5 ml of the obtained culture was inoculated in a 250-ml shake flask containing 50 ml of MEL production medium with different nitrogen sources of NaNO_3_, KNO_3_, NH_4_NO_3_, and (NH_4_)_2_SO_4_, or with different concentrations of NaNO_3_ varied from 0.5 to 4.0 g/L, or with different carbon sources of soybean oil, olive oil, glucose, and glycerol, or with different concentrations of soybean oil, varied from 50.0 to 90.0 g/L. All the cultures were aerobically incubated at 180 r/min and 28 °C for 7 days. The MEL titer, cell dry weight, and intracellular lipid content were determined as described as follows.

### Ten‑liter fed-batch fermentation

The fermentation for the MELs and intracellular lipid production was carried out in a 10-L fermentor. The seed culture of the yeast strain XM01 was prepared as described above and then inoculated with a 10% inoculation scale into 7.0 L of the initial fed-batch medium composed of 20 g/L glucose, 20 g/L soybean oil, 2.0 g/L NaNO_3_, 0.2 g/L KH_2_PO_4_, 0.2 g/L MgSO_4_·7H_2_O and 1.0 g/L yeast extract. Subsequently, depending on the consumption of soybean oil, a total volume of 60 g/L soybean oil was supplemented. The fermentation was performed under the conditions of the agitation speed of 250 r/min, the aeration rate of 400 L/h, the temperature of 28 °C, and the cultivation lasted for 192 h. During the fermentation period, the MELs titer, the content of intracellular oil, lipase activity, cell dry weight, and residual soybean oil were determined in the interval of 12 h as described as follows.

### Isolation and purification of MELs, determination of biomass, cell dry weight, intracellular lipids and residual soybean oil

MELs were extracted from the yeast culture according to a reported procedure (Rau et al. 2005b). Briefly, 30 ml of culture broth was extracted three times with an equal volume of ethyl acetate. Aqueous and organic phases were separated by centrifugation for 10 min at 8000 r/min, with the yeast cells between the two phases. The yeast cells were collected and used for the analysis of cell dry weight and intracellular lipids as reported by Wang et al. (2019). The organic phase was rotary evaporated to obtain the crude MELs, and then washed with *n*-hexane–methanol–water (1:6:3) to remove the remaining oil and fatty acids (Rau et al. 2005b). The methanol phase was collected and again rotary evaporated to obtain the purified MELs. The hexane phase was collected and rotary evaporated to determine the reducing soybean oil.

For MEL-A purification, the purified MELs were dissolved in chloroform-acetone (7:3) with a final concentration of 1.0 g/ml and applied to a silica gel column (3 cm × 40 cm) with an eluant of chloroform-acetone (7:3). The eluted samples were analyzed by thin-layer chromatography (TLC).

### Thin-layer chromatography

The MEL samples were analyzed by thin-layer chromatography (TLC) using a solvent mixture of chloroform, methanol, and NH_4_OH (65:15:2, v/v) (Niu et al. 2019). For visualization, 5% of the sulfuric acid–ethanol reagents was sprayed on the TLC plate and then heated at 80 °C for 5 min.

### HPLC analysis of hydrophilic groups and acetylation

The hydrophilic polyols and ethanoyl of MEL-A were analyzed according to a reported procedure (Wang et al. 2019). Briefly, the purified MEL-A was hydrolyzed by 2 mol/L KOH at 80 °C. The generated fatty acid was removed by chloroform extraction, and the polyols and acetic acid in the aqueous phase were analyzed by HPLC on an Agilent 1100 series HPLC system (Agilent Technologies, Palo Alto, CA, USA) equipped with an Aminex HPX-87C analytical column (300 mm × 7.8 mm) (Bio-Rad, USA).

### GC/MS analysis

To determine fatty acid composition, the purified MEL-A was transmethylated and analyzed by gas chromatography/mass spectrometry (GC/MS) as previously reported (Wang et al. 2019).

### HPLC–ESI–MS

HPLC–ESI–MS analysis of MEL-A was performed as a previously described method (Wang et al. 2019) on a Waters Acquity UPLC H-Class/SQD II system (Waters Corp., Milford, MA, USA) with an ACQUITY UPLC BEH Shield RP18 (100 mm × 2.1 mm, 1.7 μm) column. The MS detection was operated in positive ion electrospray mode with an acquisition range of *m*/*z* 50–1000 and a scan rate of 0.5 spectra/s. The mass spectral ions were identified by the calculation of elemental composition according to available literature (Goossens et al. 2016).

### Preparation and characterization of MEL nanomicelles

The purified MELs (1 mg/ml) were dissolved in deionized water and then subjected to ultrasonic treatment for 5.0 min at 260 W in an ice bath using an ultrasonicator. The sonication was carried out with the pulse function (turned on for 2 s and off for 2 s). The critical aggregation concentration (CAC) of MELs was measured with the pyrene fluorescence assay as previously reported (Sahu et al. 2011). The ratio of *I*_373_/*I*_383_ vs. Log *C* (mg/L) was plotted for the determination of the CAC value. The particle size, polydispersity index (PDI), and zeta-potential (ζ) of the prepared micelles were measured using a Zetasizer Nano ZS 90 (Malvern Instruments, UK). The morphology of this micelle was analyzed by transmission electron microscopy (TEM) observation (Yuan et al. 2021).

### Physicochemical stability assay

For storage stability analysis, the fresh MEL nanomicelles were stored at 4 °C and 25 °C for 35 days. The particle size and polydispersity index (PDI) were measured in the interval of 7 days as described above. For pH stability analysis, the pH of the MEL nanomicelles was adjusted to 1.0–13.0. The particle size, polydispersity index (PDI), and zeta-potential (*ζ*) were determined as described above.

The thermostability of the MEL nanomicelles was analyzed by differential scanning calorimetry (DSC). Briefly, thermotropic behavior of the MEL solution (1 mg/ml) and MEL nanomicelles (1 mg/ml) was determined using a McroCal PEAQ-DSC instrument (Malvern). Samples were scanned from 20 to 80 °C at a heating rate of 1 °C/min.

### Antibacterial assays

The antibacterial ability of MEL nanomicelles on the test strains of *S. aureus* (ATCC 9144), *S. epidermidis* (ATCC 12228), *P. aeruginosa* (CMCC 26069), and *E. coli* (ATCC 11775) was analyzed according to a reported protocol (Shu et al. 2019). Briefly, the tested bacterial strains of logarithmic phase with a concentration of 1.0 × 10^6^ CFU/ml were inoculated into LB broth, which was prepared with MEL nanomicelles of different concentrations (0.2, 0.5, 1.0, 2.0, 4.0, 10.0 mg/ml). LB broth without MELs was used as the control. After 12-h cultivation, the cell counting kit-8 (CCK-8) (GlpBio, USA) was used to detect the quantity of live bacterial cells and calculate the survival rate of bacteria. Minimum inhibitory concentrations (MIC) were determined as the lowest concentration of the MELs which can prevent the visible microbial growth (Shu et al. 2019).

### Clarithromycin loading by MEL nanomicelles

The clarithromycin-loaded MEL nanomicelles were prepared as previously reported (Cong et al. 2019). Briefly, the mixtures of clarithromycin (10 mg/ml in acetone) and MELs (2 mg/ml) with different final clarithromycin/MEL mass ratios of 10%, 20%, 30%, 40% (w/w) were stirred for 0.5 h at 65 °C and then were sonicated as described above for 5 min. Subsequently, the formulation was subjected to centrifugation at 12,000 r/min for 30 min to remove unloaded clarithromycin. The encapsulated amount of clarithromycin was determined using a UV spectrophotometer (210 nm) after dissolving clarithromycin-loaded micelles in an acetonitrile–water (50:50) solution. Encapsulation efficiency (EE) and loading capability (LC) were calculated as follows:

$$\mathrm{EE}\; \left(\%\right)=\frac{\text{Encapsulated clarithromycin}}{\text{The clarithromyci input}} \times 100\%,$$

$$\mathrm{LC} \; \left(\%\right)=\frac{\text{Encapsulated clarithromycin}}{\text{Weight of MELs}}\times 100\%.$$

### In vitro release profiles of clarithromycin-loaded MEL nanomicelles

The in vitro drug release profile of clarithromycin-loaded MEL nanomicelles was investigated in PBS (pH 7.4) and simulated gastric fluid SGF (pH 1.2) at 100 r/min and 37 °C, respectively, as previously reported (Cong et al. 2019). The released clarithromycin in the solution was determined at the time intervals of 1 h, 2 h, 3 h, 4 h, 6 h, 8 h, 9 h, 10 h, 12 h, 18 h, and 24 h, respectively.

## Supplementary Information

Below is the link to the electronic supplementary material.

Supplementary file1 (DOCX 1413 KB)

## Data Availability

All data generated and analyzed during this study are included in this published article and its additional files.
