# Supplementary material for: Efficient production of mannosylerythritol lipids by a marine yeast Moesziomyces aphidis XM01 and their application as self-assembly nanomicelles
Source: Mar Life Sci Technol. 2022 Jun 27;4(3):373–83. doi: 10.1007/s42995-022-00135-0 (PMC10077156; doi:10.1007/s42995-022-00135-0)
Supplement: Supplementary file 1 — Supplementary file1 (DOCX 1413 KB) [file 42995_2022_135_MOESM1_ESM.docx]

**Supplementary Information**

**Efficient production of** **mannosylerythritol lipids by a marine yeast *Moesziomyces aphidis* XM01 and their application** **as self-assembly** **nanomicelles**

Guanshuo Yu **^a,^** ^†^, Xiaoxiang Wang **^a,^** ^†^, Chao Zhang **^a^** , Zhe Chi ^a, b^, Zhenming Chi ^a, b^, Guanglei Liu ^a, b,^ *

**^a^**College of Marine Life Sciences, Ocean University of China, Qingdao 266003, China

**^b^**Laboratory for Marine Biology and Biotechnology, Pilot National Laboratory for Marine Science and Technology, Qingdao 266237, China. CAS

^†^ Guanshuo Yu and Xiaoxiang Wang contributed equally.

*Corresponding author: Guanglei Liu

E-mail: liugl@ouc.edu.cn

Tel and Fax: 0086-532-82032266


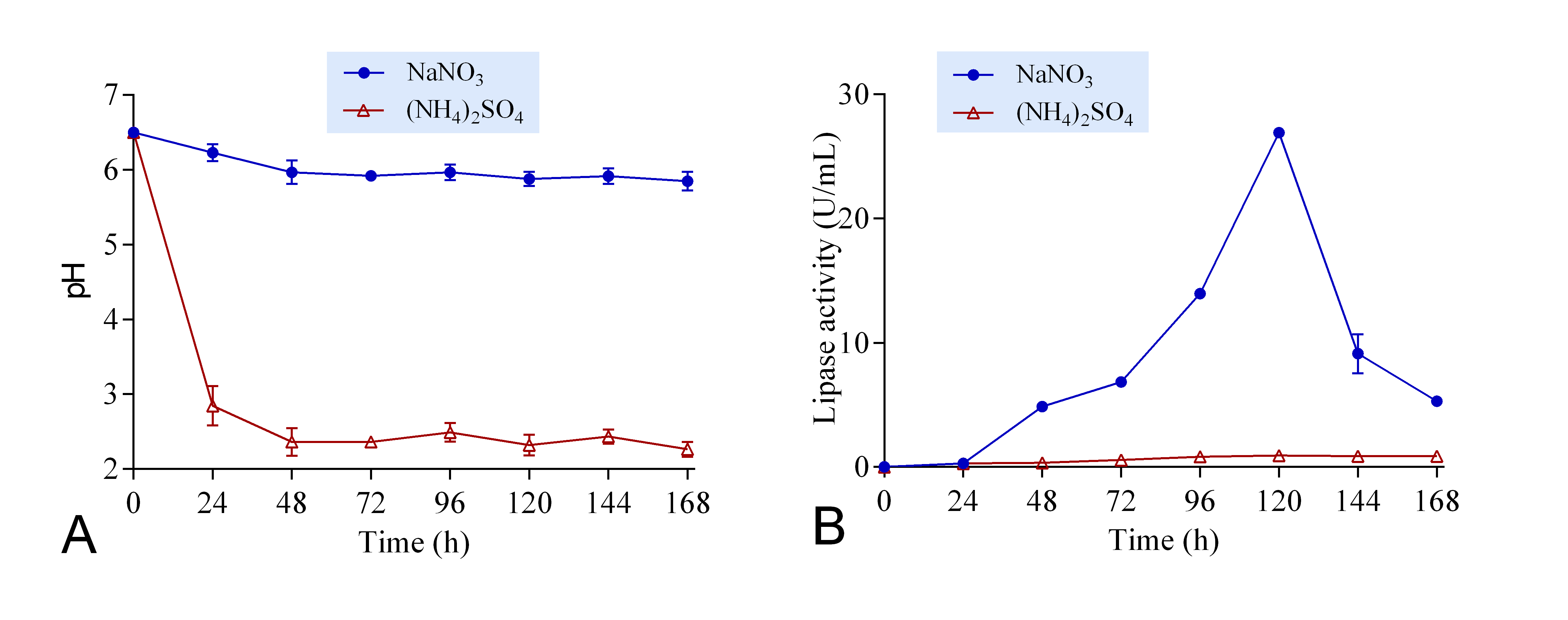


Fig. S1 Time courses of pH (A) and lipase activity (B) of *M. aphidis* XM01 cultivated in MEL production medium with NaNO_3_ or (NH_4_)_2_SO_4_ as nitrogen source. Lipase activity was determined using p-nitrophenyl palmitate (pNPP) as substrate. One unit of lipase (U) was defined as the amount of enzyme that releases 1 μmol p-nitrophenol per min at 35°C and pH 7.0.


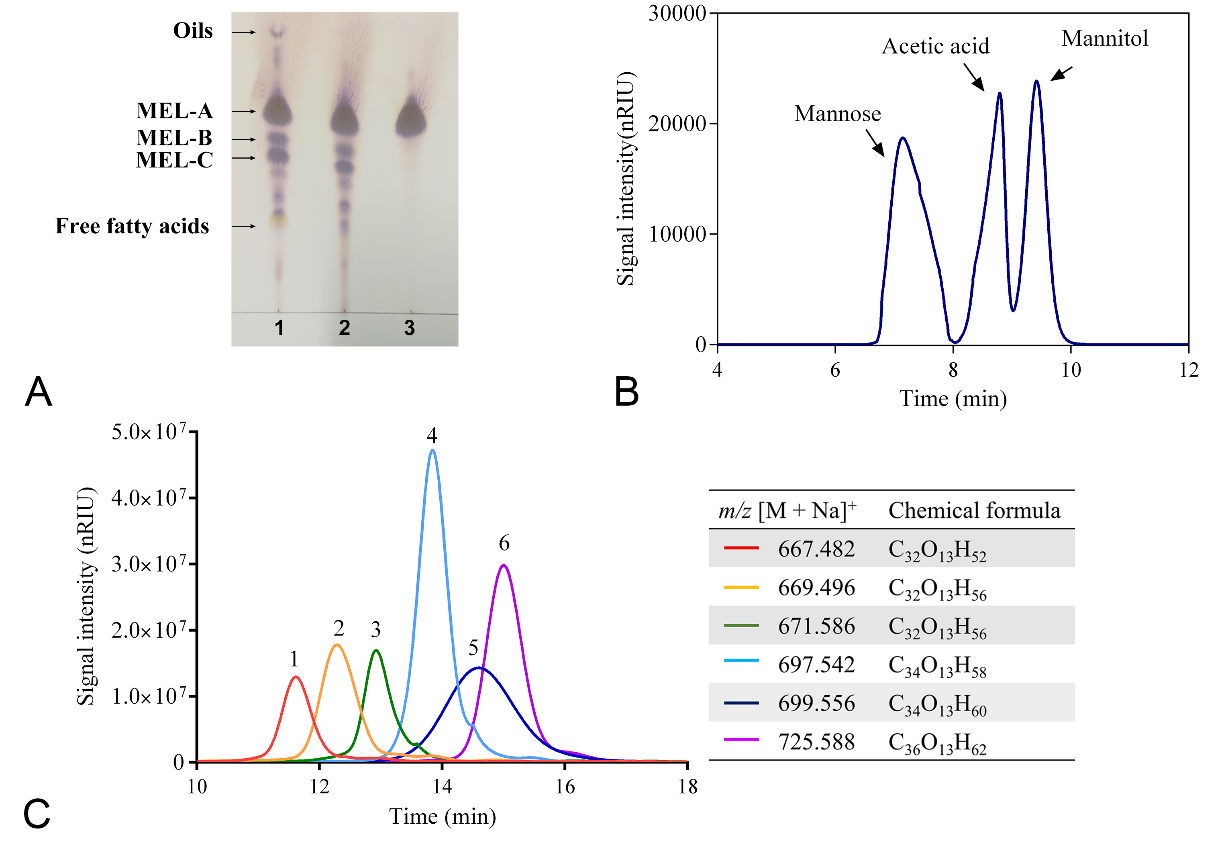


Fig. S2 Structural characterization of the MELs produced by *M. aphidis* XM01. (A) TLC of the crude MELs, the purified MELs and MEL-A. (B) HPLC analysis of mannose, mannitol, and acetic acid according to the retention time of the standard substances. (C) HPLC-ESI-MS extracted ion chromatograms of MEL-A. Based on the m/z, the fatty acid chain composition of each MEL-A peak is as follows: Peak 1: C8:1-C10:1; Peak 2: C8:0 - C10:1 or C8:1 - C10:0; Peak 3: C8:0 - C10:0; Peak 4: C10:0 - C10:1; Peak 5: C8:0-C12:0 or C10:0-C10:0; Peak 6: C10:1 - C12:0 or C10:0 - C12:1.


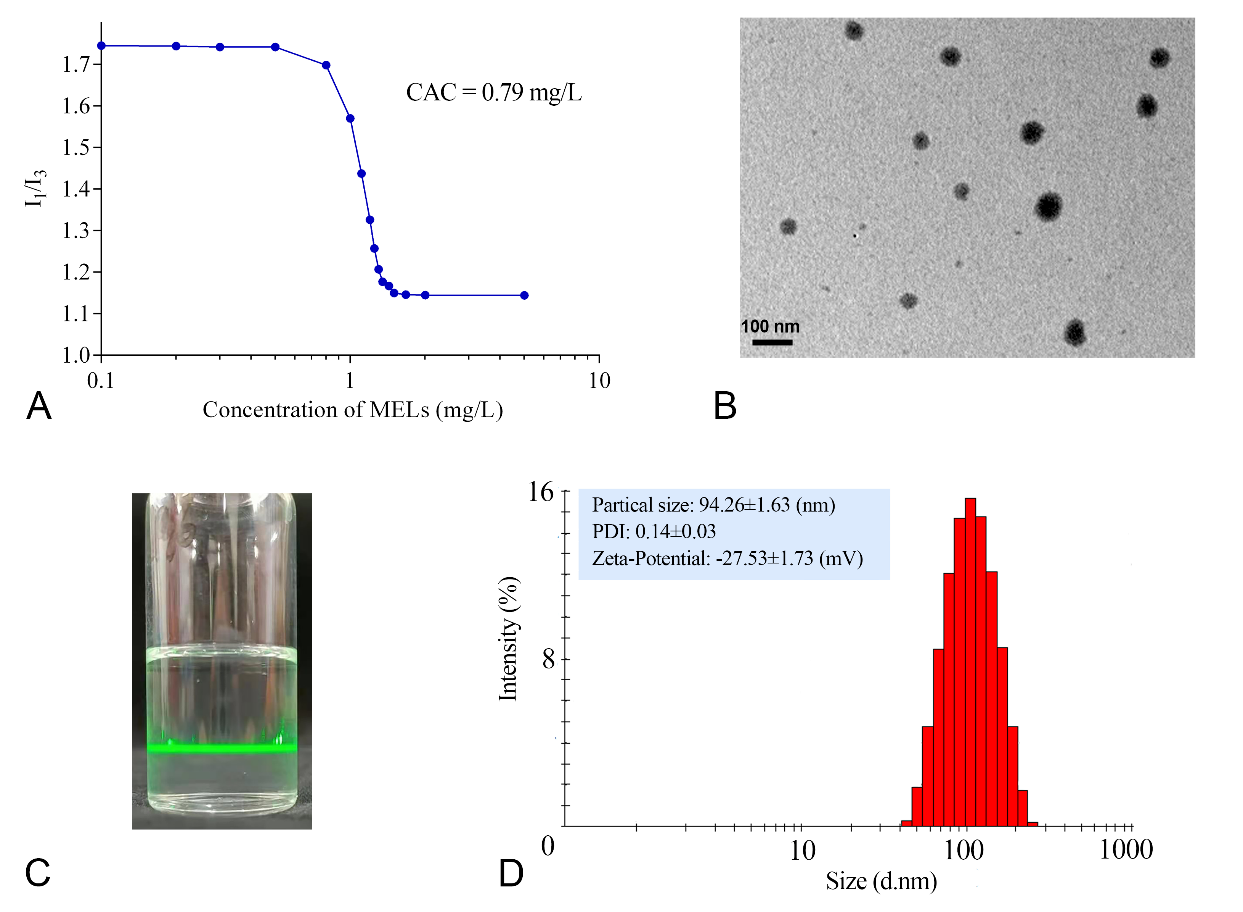


Fig. S3 Characterization of MEL nanomicelles. (A) Determination of critical micelle concentration of MEL nanomicelles by spectrofluorimetry using the pyrene probe. TEM image (B), Tyndall effect (C), and particle size distribution (D) of MEL nanomicelles.


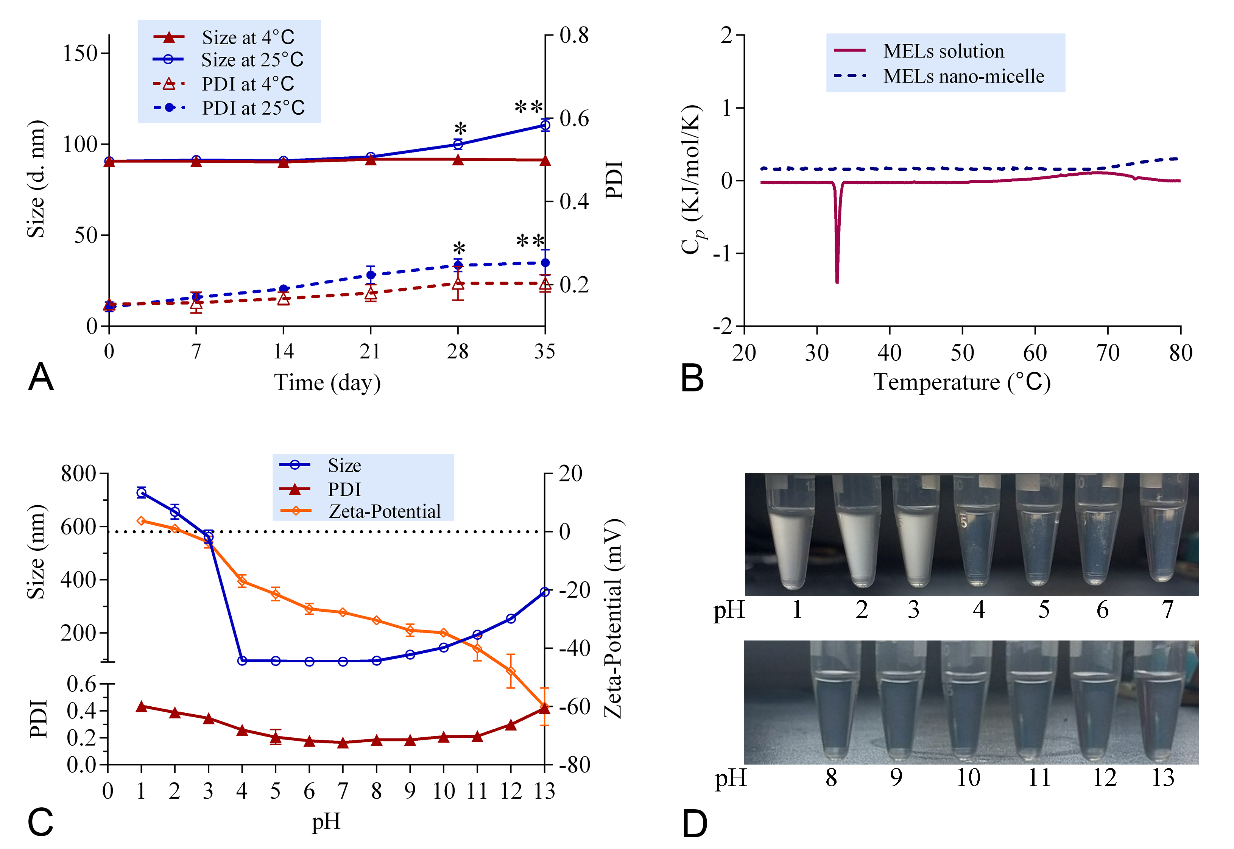


Fig. S4 Physicochemical stability of MEL nanomicelles. (A) The particle size and PDI of MEL nanomicelles at 25 °C and 4 °C after storage for 35 days. *: *P* < 0.05, **: *P* < 0.01. (B) DSC plot of MELs solution and MEL nanomicelles. (C) Effect of pH on the particle size, PDI, and zeta-potential of MEL nanomicelles. (D) Visual turbidity observation of the pH-dependent aggregation behavior of MEL nanomicelles.


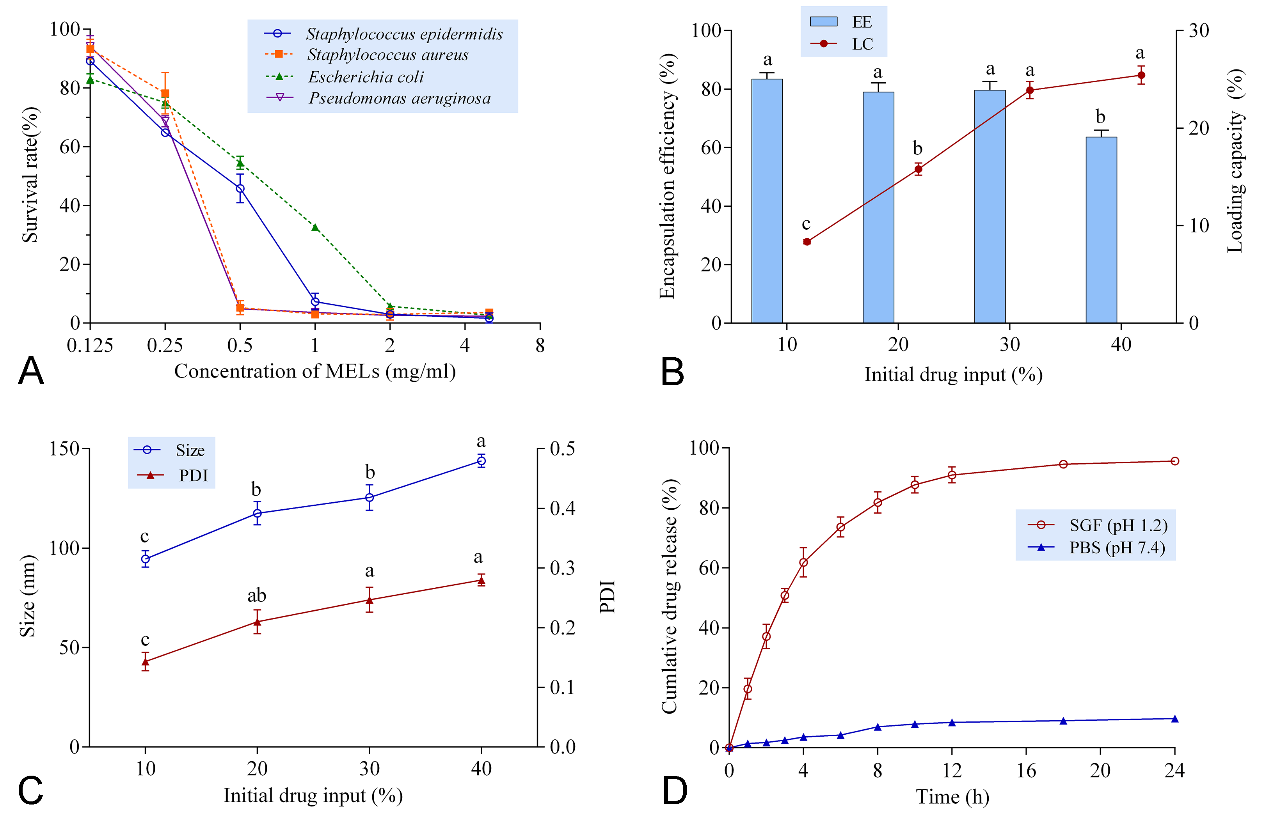


Fig. S5 Antibacterial activity and the behavior of drug encapsulation and release of MEL nanomicelles. (A) The antibacterial ability of MEL nanomicelles on the test strains of *S. aureus*, *S. epidermidis*, *P. aeruginosa*, and *E. coli*. (B) The encapsulation efficiency (EE) and loading capability (LC) of the clarithromycin-loaded MEL nanomicelles as the initial drug input varied from 0 to 40% (w/w). (C) The particle size and PDI of the clarithromycin-loaded MEL nanomicelles as the initial drug input varied from 0 to 40% (w/w). (D) The *in vitro* drug release profile of clarithromycin-loaded MEL nanomicelles in PBS (pH 7.4) and simulated gastric fluid SGF (pH 1.2). Values with different superscript letters differ significantly and are determined statistically by one way ANOVA, Tukey, *P* < 0.05.
